# Spontaneous Regression of Herniated Lumbar Disc with New Disc Protrusion in the Adjacent Level

**DOI:** 10.1155/2016/1538072

**Published:** 2016-06-26

**Authors:** Tayfun Hakan, Serkan Gürcan

**Affiliations:** ^1^The Vocational School of Health Services, Okan University, 34959 Tuzla, Turkey; ^2^Neurosurgery Clinic, International Kolan Hospital, Şişli, Istanbul, Turkey; ^3^İstanbul Gelişim University, Avcılar, 34315 İstanbul, Turkey

## Abstract

Spontaneous regression of herniated lumbar discs was reported occasionally. The mechanisms proposed for regression of disc herniation are still incomplete. This paper describes and discusses a case of spontaneous regression of herniated lumbar discs with a new disc protrusion in the adjacent level. A 41-year-old man was admitted with radiating pain and numbness in the left lower extremity with a left posterolateral disc extrusion at L5-S1 level. He was admitted to hospital with low back pain due to disc herniation caudally immigrating at L4-5 level three years ago. He refused the surgical intervention that was offered and was treated conservatively at that time. He had no neurological deficit and a history of spontaneous regression of the extruded lumbar disc; so, a conservative therapy, including bed rest, physical therapy, nonsteroidal anti-inflammatory drugs, and analgesics, was advised. In conclusion, herniated lumbar disc fragments may regress spontaneously. Reports are prone to advise conservative treatment for extruded or sequestrated lumbar disc herniations. However, these patients should be followed up closely; new herniation at adjacent/different level may occur. Furthermore, it is important to know which herniated disk should be removed and which should be treated conservatively, because disc herniation may cause serious complications as muscle weakness and cauda equine syndrome.

## 1. Introduction

Lumbar disc herniation continues to be a common health problem by decreasing the life quality and limiting the functions of the musculoskeletal system. Medical or surgical treatment can be chosen according to the clinical signs and symptoms of the patients. In some cases, spontaneous regression of herniated lumbar disc, protruded, extruded, or sequestrated, can be seen. It is a well-known phenomenon since Guinto et al. [1] demonstrated it as the first time in 1983. Examples of this rare condition, disappearance of herniated discs, were reported occasionally [2–4]. The symptoms of the patients may improve and they may return to their active life with the spontaneous absorption of the disc material. Spontaneous regression of lumbar disc herniation and a new disc protrusion in adjacent level is an exceptional condition that was reported only once in the literature [5]. Recurrence or reherniation of intervertebral disc is a common complication; but a new herniation in different segment without any recurrence is unusual. It is clear that the underlying mechanisms of disc herniation and resorption processes are very complex; as the factors cause a new disc herniation in adjacent level, the regeneration and/or reparation systems of the previously disturbed spine segment resist strongly reherniation.

Here, an additional case of a patient who experienced a new extruded lumbar disc herniation following the resorption of the previous herniation at the adjacent level is presented.

## 2. Case Presentation

**Figure 1:**
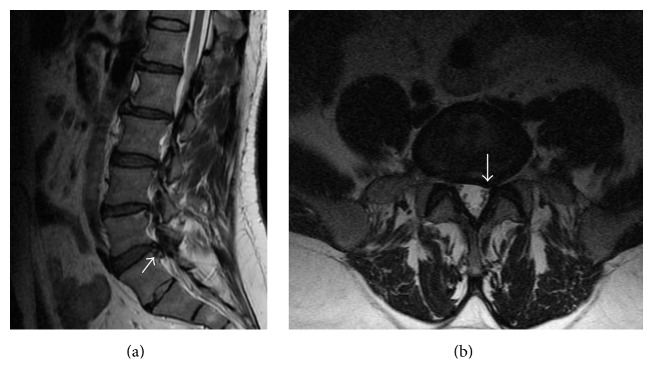
An extruded disc herniation that slightly migrated downward at L5-S1 level on T2 weighted sagittal (a) and axial (b) MR images after 3 years.

**Figure 2:**
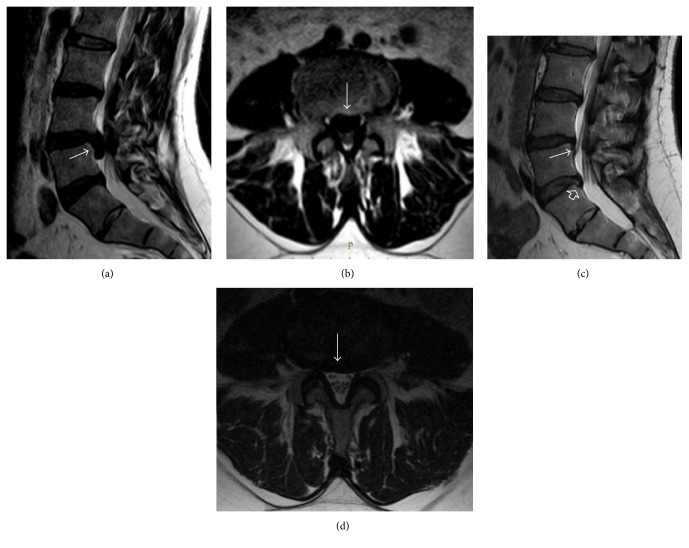
(a) A huge extruded disc herniation that migrated caudally (white arrow) at L4-5 level on T2 weighted sagittal lumbar MR images. (b) Axial view of the same disc, extruded disc occupying nearly half of the spinal canal. (c and d) The extruded disc has disappeared completely (white arrows) in sagittal and axial MR images after three years; the height of the disc space slightly decreased at L4-5 level and there was no sign of compression. The protrusion of L5-S1 disc (arrow head) that will be obvious in the left sagittal images.

A 41-year-old man was admitted with ten-day history of radiating pain and numbness in the left lower extremity. Neurological examination showed no abnormality except a positive left straight leg raising test. The Visual Analog Scale (VAS) for pain was noted as seven. Lumbar magnetic resonance imaging (MRI) revealed a left posterolateral disc extrusion at L5-S1 level (Figure 1). He said that he was admitted initially to another hospital with low back and radiating pain in both of his legs three years ago. According to him, his pain was nearly the same as that of the previous admission where he experienced low back and radiating pain in both of his legs three years ago; but he had low back and radiating pain only in his left leg for this time. Unfortunately, we were not able to reach his previous neurological records; but a huge extruded lumbar disc herniation that was caudally immigrating was found at L4-5 level when his initial MRI was examined (Figure 2). He had refused the surgical intervention that was offered and was treated conservatively with bed rest, nonsteroidal anti-inflammatory drugs, and analgesics nearly for three months at that time by our colleagues. He said that he was nearly symptom-free until the onset of new low back and left leg pain approximately for two and half years. The patient was a tradesman working on a table and defined no remarkable heavy physical stress upon a few months previous to his second involvement. A conservative therapy, including bed rest, physical therapy, nonsteroidal anti-inflammatory drugs, and analgesics was advised, because he had no neurological deficit and a history of spontaneous regression of the extruded lumbar disc.

## 3. Discussion

Lumbar disc herniation is one of the most common causes of low back pain and/or extremity radicular syndrome. Conservative management, including bed rest, oral anti-inflammatory and analgesics, spinal anesthetic blocks, and/or physical therapy, is recommended for treatment of lumbar disc herniations [6]. In the absence of symptom resolution in two months, or presence of cauda equine syndrome, muscle weakness, or progressive deficit while being medically managed, surgical intervention is advised [7]. The great potential for regression of disc herniations has been occasionally reported [2, 4–6] and it leads to questioning of the choosing the treatment modality.

Although extensive documentations are found in the literature, the mechanisms proposed for regression of disc herniation are still incomplete [4, 8]. Dehydration within the nucleus pulposus and shrinkage, a mechanical retraction of herniated material back into the annulus fibrosus, and enzymatic degradation and phagocytic reduction via immunohistologic mediators are three popular mechanisms assumed in the literature. The second mechanism, mechanical retraction of the herniated disc, is a theoretical assumption expected to occur when the disc herniation protrudes through the annulus fibrosus by protecting anatomical relation. Third mechanism which has been studied by many authors depends on a series of inflammatory responses of autoimmune system, including neovascularization, production of matrix proteinase, increasing of cytokine levels, enzymatic degradation, and macrophage phagocytosis [1, 4, 6, 9, 10].

A mass of intervertebral disc herniation may be classified as protruded, extruded, or sequestrated that represents free fragments. Sequestrated and/or large disc fragments were found to be the most regressed herniations. [4, 6, 8]. Epidural vascular supply was suspected to be an important role for regression of the extruded disc fragments through the ruptured posterior longitudinal ligament. Splendiani et al. [11] reported that herniations with high signal intensity on T2 weighted MRI sequences and free fragment were regressed in 85.18% and 100% of the cases. However, in the presented study, the herniated disc was not a free fragment and it did not have high signal density, and it had a thick anatomical relation with the intervertebral disc material.

Gürkanlar et al. [5] reported spontaneous regression of two lumbar herniations at different levels and times in the same patient. The extruded L5-S1 disc was spontaneously regressed in three years and the extruded disc was regressed after one year. In the presented study, the new herniation has occurred in the inferior level after three years. In both of these cases, spine repaired and protected the previously herniated disc side so strongly that the factors leading to new herniations in adjacent levels did not affect them.

## 4. Conclusion

It is very important to know which herniated disk should be removed, because lumbar disc herniation is not purely innocent. Gene therapy, growth factor injection, cell-based therapies, and tissue engineering approaches are among the novel strategies developed for degeneration and regeneration problems of the intervertebral disc [12]. In the future, this sort of research for intervertebral disc regenerative therapies may contribute to understanding of the mechanisms underlying the regression of protruded disc herniation and may help choose the appropriate treatment for the patients. Until that time, it is still wise to wait for surgical treatment in patients with herniated lumber disc diseases that have nearly normal neurological findings and tolerable pain.

## References

[1] Guinto F. C., Hashim H., Stumer M. (1984). CT demonstration of disk regression after conservative therapy. *American Journal of Neuroradiology*.

[2] Gezici A. R., Ergün R. (2009). Spontaneous regression of a huge subligamentous extruded disc herniation: short report of an illustrative case. *Acta Neurochirurgica*.

[3] Martínez-Quiñones J. V., Aso-Escario J., Consolini F., Arregui-Calvo R. (2010). Spontaneous regression from intervertebral disc herniation. Propos of a series of 37 cases. *Neurocirugia*.

[4] Orief T., Orz Y., Attia W., Almusrea K. (2012). Spontaneous resorption of sequestrated intervertebral disc herniation. *World Neurosurgery*.

[5] Gürkanlar D., Aciduman A., Koçak H., Günaydin A. (2005). Spontaneous regression of lumbar disc herniations at different levels and times in a patient: A case report. *Turkish Neurosurgery*.

[6] Macki M., Hernandez-Hermann M., Bydon M., Gokaslan A., McGovern K., Bydon A. (2014). Spontaneous regression of sequestrated lumbar disc herniations: literature review. *Clinical Neurology and Neurosurgery*.

[7] Rahmathulla G., Kamian K. (2014). Lumbar disc herniations ‘to operate or not’ patient selection and timing of surgery. *Korean Journal of Spine*.

[8] Kim E. S., Oladunjoye A. O., Li J. A., Kim K. D. (2014). Spontaneous regression of herniated lumbar discs. *Journal of Clinical Neuroscience*.

[9] Geiss A., Larsson K., Rydevik B., Takahashi I., Olmarker K. (2007). Autoimmune properties of nucleus pulposus: an experimental study in pigs. *Spine*.

[10] Tsarouhas A., Soufla G., Katonis P., Pasku D., Vakis A., Spandidos D. A. (2011). Transcript levels of major MMPs and ADAMTS-4 in relation to the clinicopathological profile of patients with lumbar disc herniation. *European Spine Journal*.

[11] Splendiani A., Puglielli E., De Amicis R., Barile A., Masciocchi C., Gallucci M. (2004). Spontaneous resolution of lumbar disk herniation: predictive signs for prognostic evaluation. *Neuroradiology*.

[12] Molinos M., Almeida C. R., Caldeira J., Cunha C., Gonçalves R. M., Barbosa M. A. (2015). Inflammation in intervertebral disc degeneration and regeneration. *Journal of the Royal Society Interface*.

